# Impacts of Cereal Ergot in Food Animal Production

**DOI:** 10.3389/fvets.2016.00015

**Published:** 2016-02-25

**Authors:** Stephanie Coufal-Majewski, Kim Stanford, Tim McAllister, Barry Blakley, John McKinnon, Alexandre Vieira Chaves, Yuxi Wang

**Affiliations:** ^1^Faculty of Veterinary Science, School of Life and Environmental Sciences, University of Sydney, Sydney, NSW, Australia; ^2^Lethbridge Research and Development Centre, Agriculture and Agri-Food Canada, Lethbridge, AB, Canada; ^3^Agriculture Centre, Alberta Agriculture and Forestry, Lethbridge, AB, Canada; ^4^Department of Veterinary Biomedical Sciences, University of Saskatchewan, Saskatchewan, SK, Canada; ^5^Department of Animal and Poultry Science, University of Saskatchewan, Saskatchewan, SK, Canada

**Keywords:** ergot alkaloids, sclerotia, *Claviceps purpurea*, toxicoses, animal performance

## Abstract

The negative impacts of ergot contamination of grain on the health of humans and animals were first documented during the fifth century AD. Although ergotism is now rare in humans, cleaning contaminated grain concentrates ergot bodies in screenings which are used as livestock feed. Ergot is found worldwide, with even low concentrations of alkaloids in the diet (<100 ppb total), reducing the growth efficiency of livestock. Extended periods of increased moisture and cold during flowering promote the development of ergot in cereal crops. Furthermore, the unpredictability of climate change may have detrimental impacts to important cereal crops, such as wheat, barley, and rye, favoring ergot production. Allowable limits for ergot in livestock feed are confusing as they may be determined by proportions of ergot bodies or by total levels of alkaloids, measurements that may differ widely in their estimation of toxicity. The proportion of individual alkaloids, including ergotamine, ergocristine, ergosine, ergocornine, and ergocryptine is extremely variable within ergot bodies and the relative toxicity of these alkaloids has yet to be determined. This raises concerns that current recommendations on safe levels of ergot in feeds may be unreliable. Furthermore, the total ergot alkaloid content is greatly dependent on the geographic region, harvest year, cereal species, variety, and genotype. Considerable animal-to-animal variation in the ability of the liver to detoxify ergot alkaloids also exists and the impacts of factors, such as pelleting of feeds or use of binders to reduce bioavailability of alkaloids require study. Accordingly, unknowns greatly outnumber the knowns for cereal ergot and further study to help better define allowable limits for livestock would be welcome.

## Introduction

Mycotoxigenic fungi have the ability to inhabit grain cereals, leading to decreased grain yield and quality, mycotoxin production, and reduced animal performance (1, 2). Grain ergot is found worldwide and most commonly under conditions where flowering crops are exposed to extended cold and wet periods, as ergot infects the open floret (3, 4). Ergot alkaloids are produced by a group of fungi of the genus *Claviceps* and are one of the six major classes of mycotoxins (others being aflatoxins, trichothecenes, fumonsins, zearalenone, and ochratoxins) frequently found in cereal grains. Ergot alkaloids are toxic to humans and animals if they are consumed in sufficient amounts, causing a disease called “Ergotism” (5). In most countries, grain that is contaminated with ergot is banned from human consumption and redirected for use as livestock feed (6). Consequently, ergot alkaloids continue to be a concern for livestock as allowable limits are less rigorous for feeds and the screenings containing ergot bodies are frequently used as feed. There is also a common misconception that livestock are less sensitive than humans to ergot alkaloids (7). The study of ergot toxicoses is further complicated due to climate-dependent fluctuations in fungal populations as well as genetic changes in fungi that can alter the concentration and types of alkaloids produced, potentially leading to previously uncharacterized alkaloids (7, 8). Therefore, increasing concentrations of ergot in feed grains pose a challenge for both grain and livestock industries. This review aims to describe the major ergot alkaloids currently identified in grain, how the alkaloids impact livestock and the technologies that can be used to measure alkaloids and reduce their impacts on livestock.

## Ergot and Its Lifecycle

Ergot found in grain crops arises from a parasitic fungus of the genera *Claviceps* with *Commiphora africana, Claviceps sorghi, Calotropis gigantea*, or *Claviceps purpurea*, being members and *C. purpurea* the predominant species (Table 1). The term “*purpurea*” originates from its ability to replace kernels in grain with hard purplish ergot bodies (sclerotia) that contain a diversity of alkaloids (9, 10).

**Table 1:** **Species of Claviceps found on grain crops (10)**.

Claviceps species	Host crops
*C. africana* and *C. sorghi*	Sorghum
*C. gigantea*	Maize
*C. purpurea*	Barley, wheat, rye, oats

Field and storage mycotoxins have become more abundant over the past 5 years in some areas of Canada because environmental conditions favored growth of mycotoxigenic fungi (11). For example, as much as 20% of the wheat produced in western Canada in 2011 was infected to some degree by ergot (12). With climate-change models predicting increased precipitation and prevalence of insects, concentrations of ergot in Canadian cereal grains are likely to increase in the future (13).Susceptibility of grains to ergot (from most to least) is ranked rye (*Secale cereale*), wheat (*Triticum spp*.), triticale (*Triticosecale*), barley (*Hordeum vulgare*), and oats [*Avena sativa*; (14)]. Rye, an open pollinator is more susceptible to ergot infection, whereas wheat and barley are self-pollinators. Ergot contamination typically reduces yield by 5–10% (rye and wheat, respectively), but the reduction in quality grade accounts for the majority of the economic loss associated with contaminated grain (14). Ergot alkaloids are also produced by the fungus *Neotyphodium coenophialum* in grasses, particularly fescues (15). Fescue toxicosis is prevalent in the costal and tableland regions of Australia and is estimated to cost ranchers in the USA more than $860 million per year (16, 17).

The life cycle of ergot has two stages, germination and the honeydew stage (9). While germination typically refers to the developmental stage from a seed to plant growth, ergot germination is defined by drumstick-shaped fruiting structures that develop from the sclerotia (9). These structures produce spores known as ascospores, which become wind-borne and easily infect the ovaries of flowering cereals (9). Contaminated grain heads can contain multiple ergot sclerotia that often require differing incubation periods to germinate. Generally, the sclerotia of *C. purpurea* require 4–8 weeks at 0–10°C to initiate germination, with higher temperatures (>25°C) prolonging germination (18). The optimal temperature range for germination of ergot in rye is thought to be 18–20°C (19), although germination in rye has also been documented between 9 and 15°C (20). Furthermore, it has been noted that germination can occur without a chilling period, but ergot body formation is enhanced during cool, wet weather, especially during the flowering stage (19).

The second stage involves the florets oozing a sticky conidia that is spread by insects and in moist environments. Following the honeydew stage, the infected ovary hardens and is replaced by an ergot body that either falls before or during harvest, contaminating the field or the harvested grain (21). However, if the flowers had fertilized prior to infection, they would have become resistant (10).

## Ergot Alkaloids

Although fescue toxicoses have been studied for over 50 years, the alkaloids prevalent in fescue differ from those in grain (22) and few studies have investigated the impact of grain ergot on livestock production (23). Cattle, sheep, and swine have a greater tolerance of mycotoxins produced by *Fusarium* spp. such as deoxynivalenol (DON) than for ergot alkaloids (24, 25). The FDA restricts the levels of DON in grains and grain by-products to 5 ppm for swine and 10 ppm for cattle as greater concentrations can adversely impact weight gain (26).

Concentrations of ergot alkaloids in the sclerotia of *Claviceps* can be as great as 0.75% DM (27). The concentration and the type of alkaloid produced can vary among fungal species, the type of cereal grain and with environmental conditions, with production being more pronounced in periods of heavy rainfall and with moist soils (10, 28). More than 50 different ergot alkaloids have been identified in grains infected with *Claviceps spp*., which are divided into ergopeptine and ergoline alkaloid subfamilies. These are further divided into three biogenetically related classes: clavinet, simple sysergic acid derivatives, and peptide alkaloids [Table 2; (10, 29)]. However, new alkaloids are continually discovered further increasing the complexity of defining the toxicity of ergot (29).

**Table 2:** **Limits of detection (LOD) and retention time of major ergot alkaloids and their epimers in wheat flour (30)**.

Ergot alkaloid	LOD (μg/g)	Retention time (min)
Ergometrine	0.0034	6.6
Ergometrinine	0.0017	7.2
Ergotamine	0.0093	8.2
Ergotaminine	0.012	9.8
Ergosine	0.0063	8.1
Ergosinine	0.0030	9.5
Ergocristine	0.017	9.1
Ergocristinine	0.021	10.5
Ergocryptine	0.0023	9.0
Ergocryptinine	0.0081	10.4
Ergocornine	0.0060	8.7
Ergocorinine	0.0055	10.1

The most dominant alkaloids in grain ergot bodies are ergotamine, ergocristine, ergosine, ergocornine, and ergocryptine (29). By contrast, ergovaline is the most common form of alkaloid present in forages infected by endophytic fungi, followed by ergine (3, 31, 32). Endophytic fungi produce alkaloid concentrations far lower than those found in the sclerotia of *Claviceps*, accounting for the differences in clinical symptoms between the two forms of toxicoses (7).

**Figure 1:**
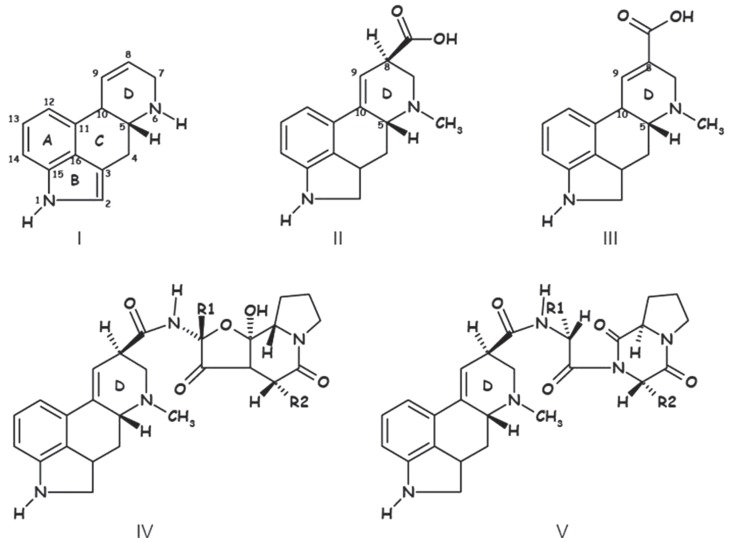
**Chemical structure of ergoline (I), lysergic acid (II) paspalic acid (III) ergopeptines (IV), and lactam ergot alkaloids – ergopeptams (V) (35)**.

**Figure 2:**
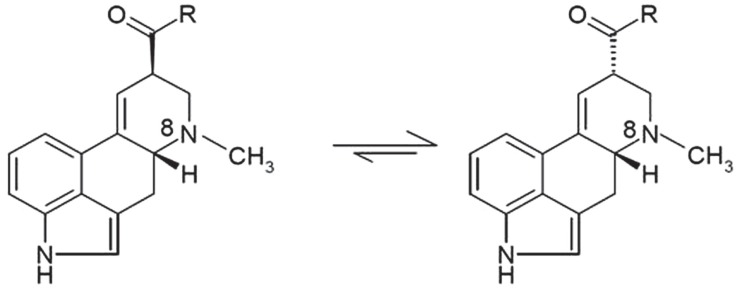
**Ergot alkaloids containing C9 = C10 double bond readily epimerize at the center of symmetry C-8, adapted from Crews (36)**.

When describing ergot alkaloids, it is essential to identify their chemical structure [Figure 1; (27)] as the degree of toxicity may be dependent on the nature of the matrix and feed processing technique. The main ergot alkaloids, such as ergometrine, ergotamine, ergosine, ergocristine, ergocryptine, and ergocornine, are structurally similar, differing only in substitutions on C-8 (13). Moreover, alkaloids containing a C9 = C10 double bond easily epimerize depending on temperature and pH [Figure 2; (10, 33)] and it is possible that application of heat during pelleting may alter chemical bonds and the chemical composition of feed (34). However, minimal effects on ergot alkaloids have been observed at storage temperatures <5°C (33), but prolonged storage at higher temperatures can increase the amount of ergopeptinines that arise from natural right-hand rotation epimerization [C-8-(S); (10)].

The activation of “-ines” to “-inines” is rapid in acidic and alkaline solutions, increasing the challenge of ergot removal using extraction and cleaning processes. Avoiding the reactivation of -ines is important as this conversion appears to produce products that are more toxic to livestock (10, 37).

## Determination of Ergot and Ergot Alkaloids

Analytical methods to determine ergot alkaloids should aim to detect major alkaloids in combination with their corresponding biologically active metabolites. While some techniques are more sensitive than others, European Feed Standard Association (EFSA) determined that new validated methods are still required to quantify ergot alkaloids in feed materials to provide more reliable regulatory limits for each individual alkaloid in food and feed (27). All methods have detection limits, yet information concerning these limits for different alkaloid types is scarce.

### Ergot Contamination by Visual Detection

Ergot is typically detected upon visual inspection, with dark sclerotia bodies being up to 10 times larger than grain kernels. However, ergot bodies may range in size from a few millimeters to more than 4 cm depending on the size of the host plant (10). In some cases, sclerotia bodies are smaller (21), increasing the degree of difficulty in detecting them within grain screenings (25). Upon visual inspection, counting >5 sclerotia/L grain, or having sclerotia weighing 0.1–0.3% of grain DM is sufficient contamination that the grain should not be fed to pregnant or lactating livestock (38).

### Thin-Layer Chromatography

This method uses a plate that is coated in a solid adsorbent (silica gel) in combination with a small amount of the mixed sample to be analyzed (39). The method is often used to identify a compound of interest in a mixture, as different components will vary in solubility and, therefore, migrate and be absorbed at different locations on the plate. Lobo et al. (40) found that it was difficult to separate the 12 main alkaloids in rye ergot, even using two-dimensional thin-layer chromatography (TLC), a result that likely reflects the low sensitivity of the method (7). Nevertheless, TLC may be valuable for separating individual alkaloids, particularly in developing countries (10, 35).

### Liquid Chromatography and Mass Spectrometric Detection

Liquid chromatography (LC) is often used to analyze ergot alkaloids in combination with mass spectrometric detection (MS) for different matrixes in feed and foodstuffs (35). The benefit of this technique is that any known alkaloid can be determined in one run using solvent extraction, separation, detection, and quantification (10, 38).

Although only a few studies have used LC–MS–MS to detect ergot alkaloids, this technique is useful for structural confirmation and the identification of unknown alkaloids (10). Stahl and Naegele (41) reported that this technique can be used to reveal unknown ergot derivatives (semi-synthetically-derived alkaloids, such as lysergic acid diethylamide), emphasizing the importance of implementing such chemical analysis for future research. Blakley and Cowan (38) described quantification of four common ergot alkaloids using this method and recognized that combined alkaloid content should not exceed 100–200 ppb (60 g of grain is required for analysis). However, issues with the collection of representative samples, variation in kernel size, and crop type may produce inaccurate results with this method (38).

**Figure 3:**
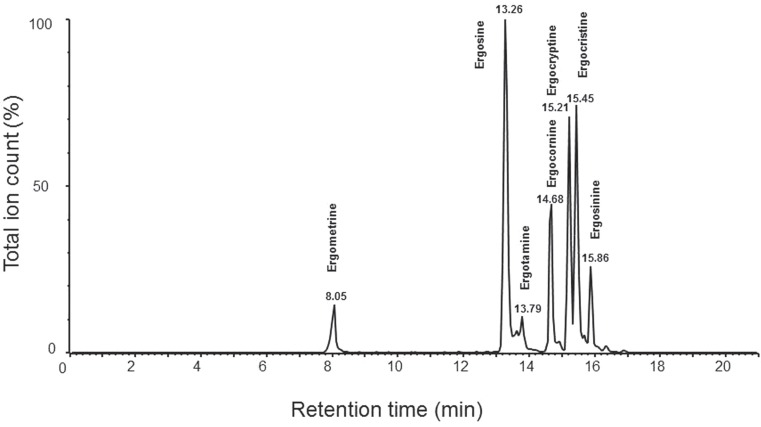
**Retention times of ergometrine (8.05 min), ergosine (13.26 min), ergotamine (13.79 min), ergocornine (14.68 min), ergocryptine (15.21 min), ergocristine (15.45 min), and ergosinine (15.86 min) from LC–MS consisting a Agilent 1100 HPLC system with Agilent Zorbax Eclipse XDB-C18 narrow bore 2.1 mm × 150 mm, 5 μm HPLC column and a Quattro Ultima Pt mass spectrometer**. The analysis uses mixture of acetonitrile (85%) and 10 mM ammonium acetate (15%) as sample extraction solvent and 10 mM ammonium acetate as mobile phase A and acetonitrile as mobile phase B with the same analytic conditions as described by Krska et al. (42).

Byrd (30) determined the limits of detection (LOD) of six ergot alkaloids in wheat and their epimers, in combination with their corresponding retention time (Table 2). Moreover, Krska and Crews (10) validated the use of LC–MS as a means of reliable detection in determining certain alkaloids, yet today only six alkaloids and their isomers can be accurately identified using this method (Figure 3).

### High-Performance Liquid Chromatography

High-performance liquid chromatography (HPLC) uses a column to pump the sample mixture at great pressure in a solvent with chromatographic packing material, producing excitation wavelengths ranging between 235 and 250 nm as detected by UV absorption (43, 44). With the ability to detect compounds at concentrations as low as parts per trillion, HPLC is a common method currently used to identify ergot alkaloids. The most common alkaloids detected using this method are ergometrine, ergotamine, ergocornine, ergocryptine, ergocristine, ergosine, and their respective isomers, with the sum of these alkaloids equating to total alkaloid content (45).

Although alkaloid concentrations have been detected as low as 0.02–1.2 μg/kg using multi-analyte LC–MS/MS, extensive epimerization was noted, affecting the estimation of overall alkaloid content (10). Sulyok et al. (46) demonstrated that HPLC could detect concentrations as low as 0.17–2.78 μg/kg without epimerization, validating the prevalent use of HPLC for determining alkaloid content.

### Enzyme-Linked Immunosorbent Assay

The enzyme-linked immunosorbent assay (ELISA) involves combining antibodies with an enzyme-mediated color change (commonly alkaline phosphatase and glucose oxidase) to identify small quantities of targeted substances. The antigen is capable of binding to the specific antibody, which can be identified by a secondary antibody and revealed using fluorogenic substrates (47). This technique is attractive for ergot screenings in crops, but has difficulty in identifying a marker toxin to serve as a standard to determine the extent of alkaloid contamination (48). Furthermore, cross-reactivity can vary substantially depending on the nature of alkaloids being detected (10).

### Near Infrared Spectroscopy

The near infrared spectroscopy (NIR) method is used to estimate total ergot alkaloid content, particularly in tall fescue, with calibrations based on measurement obtained through ELISA (35). This method can be employed with both grain and pelleted feeds; however, pelleted grain must be ground prior to measurement to improve the accuracy of estimates (2, 35). A great advantage of NIR is the speed of detection and its ability to analyze both large and small quantities of feeds, thereby avoiding errors associated with inconsistent sampling (2). The system can also make measurements in real time by placing sensors in grain augers or belt systems (100 kg grain can be analyzed in 1 h). However, the system is heavily dependent on the establishment of an accurate calibration in which alkaloids have been measured using the sensitive techniques described above. Variation in the types of alkaloids present in grains and feeds may make development of universal calibration equations difficult.

### Detection in Animal Tissues

Alkaloids, such as ergocornine, can decrease pituitary prolactin release and counteract the stimulatory effect of estrogen on prolactin concentrations, significantly reducing milk production (49). Therefore, isolating serum from whole blood and conducting prolactin analysis may be useful in the detection of ergot alkaloids as a low prolactin concentration could be indicative of ergot alkaloid poisoning. Direct detection of ergot alkaloids and/or their derivatives in liver tissues is as yet, only at a preliminary stage (25).

Tissue accumulation of ergot alkaloids, while of concern, has been little studied, largely due to a lack of suitable assays. Dairy cattle fed 125 mg ergot alkaloids/kg dietary DM over a 2-week period led to a carry-over of toxins into milk, although less than 10% of ingested ergot alkaloids were detected (50). However, when swine were fed 1–10 g ergot/kg body weight, no evidence of ergot alkaloid residues was found in meat (45). Additional knowledge of the kinetics, metabolism, and tissue deposition of ergot alkaloids is required to determine whether the carry-over of alkaloids to livestock products other than milk occurs (35).

## Feeding Ergot-Contaminated Grain to Livestock

### Allowable Limits

The concentration of ergot alkaloids that are allowable for livestock consumption is presently contentious, as there are several different measurements in the literature that are not interchangeable. The toxicity of ergot alkaloids depends on both the type and the absolute concentration of the individual alkaloid as well as interactions with other mycotoxins that may be present in feed (27).

Individual countries have established specific tolerances for concentrations of ergot bodies in both cereal grains and animal feed (Table 3). Legislation is in place that sets the limits of ergot contamination in cereal grains for the human market at 0.05% in Australia and European Union (EU) and 0–0.05% in North America. The EU and the United States require grains destined for livestock feed to contain less than 0.1 and 0.3 ppm total ergot, respectively. The United Kingdom has a 0.001 ppm tolerance for total ergot in animal feeds. Grain exceeding these limits is banned from entering either the food or feed chain.

**Table 3:** **Allowable levels of ergot contamination (ppm) in cereal grains and feed in various regions of the world [T, triticale; W, wheat; R, rye; B, barley; O, oats; (51)]**.

Region	Ergot limit in cereal grains for humans (ppm)	Ergot limit in animal feed (ppm)	Other comments
Australia and New Zealand	0.05	N/A	0–0.1% (T)
Canada	0–0.05	0.10–0.33	Varies with grade of wheat
European Union	0.05	0.10	–
Switzerland	0.02	N/A	0.05 limit on cereals destined for milling
Japan	0.04	N/A	–
United Kingdom	Zero tolerance	0.001	–
United States	0.3 (W, R)	0.3 (W, R)	0.1% (B, O, T)

*N/A, not available*.

In Canada, maximum allowable levels of ergot alkaloids in cattle and swine feed have also been established and are 2–3 and 4–6 ppm, respectively (5). It is also recommended that feed contaminated with 250 ppb ergot alkaloids not be fed to pregnant or lactating animals due to a greater risk of abortion and agalactia syndrome. In general, 5–10 μg ergot alkaloids/kg body weight represents the general threshold dosage for all livestock (5), yet EFSA recommends doses as low as 0.6–1 μg of ergot alkaloids/kg body weight to avoid their vasoconstrictive effects (27).

Although legislation establishes tolerances for ergot alkaloids or ergot bodies in livestock feed, in most cases these concentrations have not been established through toxicological studies with livestock (2, 5, 51). For example, dietary concentrations of ergot alkaloids as low as 100–200 ppb (ergovaline) can have adverse impacts on livestock growth, especially livestock suffering from heat stress and interactions among alkaloids can lead to heightened toxicity (25). The concentration of alkaloids in the ergot bodies also varies between 0.01 and 0.21% (27). The great variation in reported impacts of ergot on animal performance has led to inconsistent recommendations of tolerable limits of ergot across countries (2). It is also evident that calves and horses are the most sensitive to ergotism, with poultry having the greatest tolerance [Table 4; (5)].

**Table 4:** **Recommended practical limits for ergot or ergot alkaloids in animal feeds to reduce negative effects on health and performance**.

Animal	Recommended ergot alkaloids practical limits [ppm; (52)]			Maximum tolerance (allowable) level of ergot alkaloids [ppm; (5)]
	Low	Moderate	High	
Piglets/sows/gilts	0.5	1	2	4–6
Poultry broiler/layer	0.75	1.5	3	6–9
Dairy/beef cattle	0.5	1	2	2–3
Calf	0.25	0.5	1	2–3
Horses	0.25	0.5	1	2–3

### Impact of Feed Processing and Grain Storage on Ergot Alkaloids

Unlike other mycotoxins that are capable of forming post-harvest as a result of spoilage during storage, ergot only forms pre-harvest, with concentrations of alkaloids remaining relatively constant during storage (25). However, Krska and Crews found that extended storage of high-moisture grain that led to aerobic instability resulted in increased ergopeptinines by promoting ergot growth (10). Despite speculations that alkaloids may degrade over time, ergot stored at 15°C for 12 months still germinated, emphasizing the importance of screening techniques to avoid propagation of ergot in grain (18). Storage temperatures lower than 5°C had little effect on ergot alkaloids (33), although high-temperature storage has the potential to alter their chemical structure and biological activity.

Pelleted grain screenings are a popular low-cost feed for both sheep and cattle. Anecdotal observations suggest that pelleting or high-temperature processing of feed may increase the bioavailability of ergot alkaloids (25), but this possibility has not been investigated experimentally. Grain by-products used for ethanol production, such as distiller’s grains, are frequently fed to livestock, although this product retains and concentrates ergot alkaloids through the production process (25). However, the effects of fermentation on the activity of ergot alkaloids and potential implication for animal health and productivity have not been fully studied. Further complications arise with grain after being processed and pelleted as ergot is then impossible to visually detect.

### Effects of Ergot Alkaloids on Health and Productivity of Livestock

#### Clinical Symptoms of Ergot Poisoning in Livestock

Ergot toxicity was first described in the middle ages as a gangrenous outbreak in humans known as “St. Anthony’s fire,” responsible for disfigurement of people and deaths (3, 7). At present, ergot poisoning rarely occurs in humans due to advanced grain processing technology and strict legislation.

Clinical symptoms of ergot poisoning can be manifested in as little as a few hours or may require months to become observable. This variability reflects differences in physiological responses to the type and concentration of alkaloids and accounts for the frequent misdiagnosis of the condition (5). Furthermore, symptoms of ergot toxicosis often resemble other conditions, such as foot rot, frostbite, and respiratory disease, further complicating diagnosis (25, 53).

Generally, ergot toxicosis is manifested in three forms:

(1) *Convulsive:* convulsions, staggering, muscle spasms, and temporary paralysis occur. This condition is often confused with tremors associated with *Claviceps paspali* (which contains great amounts of lysergic acid). This type of poisoning is more common in sheep and horses but seldom seen in cattle [Table 5; (29)]. Upon slaughter, rigor mortis is never complete, leaving muscles flaccid.
(2) *Gangrenous:* this form results in lameness, followed by the loss of extremities, such as the ears, tail, hooves, and in severe conditions even limbs (7). This form results from impaired circulation and blood supply and is most common in cattle and pigs. The condition is more severe under hot or cold conditions where vasoconstriction or vasodilation is necessary for thermoregulation (28). Gangrenous ergotism can require up to 3 months to become clinically obvious, with early symptoms, including an elevated respiration rate, gradual weight loss, a reduction in milk production, and reduced reproductive performance.
(3) *Other:* these symptoms can be less severe and include *v*omiting (enteroergotism), fever (hyperthermic ergotism), and alterations in endocrine function. Long-term exposure to ergot, intensified during hot and humid conditions, favors hyperthermic ergotism (54). Heifers injected with ergotamine and ergonovine exhibited a combination of symptoms, such as lower skin temperature, heart rate, and blood prolactin concentrations, with an increase in respiration rate and blood pressure (55). Chronic exposure to alkaloids can result in the greatest economic losses due to decreased reproductive performance and increased abortions (3).

**Table 5:** **Summary of ergot symptoms in mammals (8)**.

Form of ergotism	Species	Subfamily	Toxic alkaloid(s)	Symptoms
Convulsive ergotism	*C. purpurea*	Ergoline	Ergotoxin, ergometrine, ergotoxin (lysergic acid amines including lysergic acid, lysergol, ergine)	Writhing, tremors, twisted neck or head tilt (torticollis), confusion, hallucinations, tingling sensation underneath the skin (formication) and death
Gangrenous ergotism	*C. purpurea*	Ergopeptine (total dietary concentrations of >100–200 ppm can lead to death)	Ergotoxin, ergometrine, ergotoxin (lysergic acid amines), ergovaline, ergocryptine	Vasoconstriction, hot and cold feelings in the extremities, cold skin, spontaneous abortion, heat stress, severe lameness, reduced feed intake, reduced growth rate, agalactia, and gangrene. Ergocryptine affects prolactin levels and greatly reduces or eliminates milk production for lactation
Enteroergotism	*C. fusiformis*	Unknown	Clavine	Nausea, vomiting, somnolence, and giddiness
Hyperthermic ergotism	*A. coenophialum, C. africana, C. cyperi, C. purpurea, C. sorghi*	Unknown	Ergotamine, ergosine, and agroclavine	Fever, diarrhea, clear nasal discharge, weight loss, labored breathing, increased metabolic rate, excessive salivation, and low levels of prolactin

Ergot toxicosis can often be misdiagnosed as other forms of syndromes associated with feed refusal such as those associated with vomitoxin (56, 57).

#### Effects on Health and Performance of Livestock Animals

Consumption of ergot-contaminated grains can have negative effects on feed intake, growth, and reproduction, but factors such as livestock species, age, and the presence of other stressors such as heat or cold can influence the extent of negative health outcomes (58). Low concentrations of ergot alkaloids (<2 ppm) in feed can depress animal performance and result in intoxication, especially if feeds are administered for a prolonged period of time.

##### Animal Growth

Cattle fed diets containing 1.6% ergot (12.7 g ergot intake/day) exhibited a lower average daily gain (0.55 vs. 0.83 kg/day) and lower feed intake (6.36 vs. 10.1 kg/day) as compared to those fed uncontaminated grain (59). The study also showed that ergot intake from 1.14 to 8.17 g/day had little effect but at 12.7 g/day significantly decreased feed intake. By contrast, growth rate was linearly decreased with ergot intakes from 0 to 12.7 g/day. This observation suggests that ergot alkaloids have a direct negative impact on energy metabolism and feed efficiency when ergot intake exceeds certain limit.

##### Reproductive Performance

Cattle consuming endophytic fescue have consistently lower prolactin concentrations in plasma, with minimal changes in plasma luteinizing hormone or growth hormone [GH; (60, 61)]. Prolactin concentrations sharply declined and plateaued in cattle intravenously injected with 7 mg of ergotamine tartrate in saline over 240 min [average dosage of ergot alkaloids was 28.8 μg/kg body weight; (62)]. This decline in prolactin secretion is due to activation of D2-dopamine receptors in pituitary lactotrophs (10). Furthermore, lysergic acid derivatives are structurally similar to noradrenaline transmitters, including dopamine and serotonin, enabling ergot to disrupt the endocrine system (10).

By contrast, plasma GH concentrations in steers exhibited a transient increase after ergot alkaloid administration (23.8 μg/kg body weight) through i.v. injections (62). While ergot derivatives increased human GH concentrations, ergotamine had no impact on GH secretion from rat pituitary cells (63, 64). Accordingly, Browning et al. (62) found that cattle fed endophytic fescue displayed greater GH concentrations compared to steers grazing fescue with low endophyte content. A suppression of luteinizing hormone when ergotamine was injected suggests that this alkaloid alters the activity of the hypothalamic–pituitary–gonadal axis. By contrast, Christopher et al. (65) demonstrated that tall fescue has suppressive effects on GH secretion in ovariectomized heifers. Consequently, with acute exposure to alkaloids, particularly ergotamine or ergonovine, noticeable alterations to plasma concentrations of prolactin, GH, and luteinizing hormone become apparent (62). Similar endocrine impacts from grain ergot alkaloids are also likely, although have yet to be studied.

##### Pregnancy Rates

The alkaloids that promote vasoconstriction and lead to gangrene can also promote developmental and reproductive toxicity, such as abortions by restricting blood supply to the uterus. Duckett et al. (17) documented that ewes fed endophyte-infected tall fescue seed had shorter gestation lengths (up to 5-day difference), leading to a 2 kg reduction in lamb birth weights. During pregnancy, consumption of ergot alkaloids can impact maternal lipid metabolism, mammary growth and reduce milk production and secretion from the inhibition of prolactin release (17, 66). Similarly, compared to cows consuming endophyte-free fescue, Watson et al. (67) observed a 15% reduction in birth weight of calves delivered from cows consuming endophyte-infected fescue. However, both occurred under high ambient temperatures conditions where alkaloid consumption has the greatest impact on reproductive function. As umbilical blood flow increases throughout pregnancy, the vasoconstrictive response to ergot alkaloids can restrict blood flow to the fetus and impair fetal development (17). Moreover, Dyer (68) also observed that ergovaline induced contraction in the uterus further altering fetal development.

Abortions and premature births have been noted in sows fed grain ergot (69). Similarly, supplementing ewes with 0.1, 0.5, or 0.7% ergot-contaminated feed decreased lambing by 20% (70). However, because there were no data on the type of ergot and the quantities of alkaloids in these studies, it is difficult to determine whether the reduced pregnancy rate was due to ergot or other mycotoxins. In a later study, Burfening (59) reported that lambing rate increased to 0.87 lambs/ewe when ewes were fed diet containing 0.5% ergot. This was contrasted to the observation that the lambing rate declined from 1.02 to 0.78 lambs/ewe when ewes were fed diets containing 0.1% ergot. This may demonstrate adaptability of the ewes to the toxin throughout pregnancy or differences in relative concentrations of alkaloids in the two studies. Furthermore, lambs fed a 0.5 or 0.7% ergot-contaminated diet demonstrated a greater susceptibility to lameness with 21% of lambs showing signs of impaired movement. However, no abortions were observed in either of the above trials, although reduced body condition from grain ergot ingestion was noted (70).

Agalactia refers to the absence or failure to secrete milk, displaying irreversible effects for pregnant livestock during late gestation, with greatest susceptibility in sows (71). A direct correlation with a decrease in prolactin secretion and the inhibition of milk production was first identified by Zeilmaker and Carlsen (72) in rats injected with 1 mg of ergocornine, a condition that could be reversed by continuous administration of prolactin. Similarly, it has been shown that feeding 0.5–1.0% ergot to gestating sows impaired udder development (73). Yaremcio (74) proposed that estrogen concentrations in ergot can cause abortions, along with temporary sterility resulting in lowered subsequent conception rates. Hence, even low concentrations of ergot should be avoided in the feed of pregnant or lactating animals to avoid the risk of underdeveloped neonates and reduced mammary tissue development (71). However, even though several studies have showed that prolactin concentrations decrease upon exposure to ergot alkaloids (49, 75), milk production in ewes fed diets containing 0.5–0.7% grain ergot did not decrease (70).

##### Sperm Motility

Some ergot alkaloids can negatively affect sperm and uterine motility in mammals through agonistic interactions with dopaminergic, alpha-adrenergic, and serotonergic receptors (76, 77). Such membrane receptors are involved in the regulation of mammalian sperm function and increases in intracellular cAMP and calcium concentrations can negatively impact the motility of bovine spermatozoa (77). Moreover, ingestion of ergot alkaloids by growing bulls depressed growth rate, serum prolactin concentration, scrotal circumference, and sperm motility (78). Treating sperm with ergonovine (20 mg/mL) resulted in the greatest reduction in sperm motility and the percentage of intact acrosomes as compared to treatment with phenylephrine, oxytocin, and norepinephrine (76). Ultimately, sperm motility is affected by grain ergot, whereas both cortisol and testosterone concentrations are not impaired when bulls were fed toxic endophyte-infected and novel endophyte-infected feed (78). It has been shown that the interaction of ergot alkaloids with membrane receptors is complex and different alkaloids affect different receptors in different types of tissues (77).

### Species Differences

In addition to noticeable differences in tolerance levels between species, variation in absorption rate and ability to detoxify toxins is extremely diverse. Although poultry is regarded as a group for recommended allowable limits, dependent on species, they can either be quite tolerant or extremely sensitive (ducks) to ergot alkaloids. This demonstrates the need to develop recommended allowable limits of alkaloids for all species of livestock and poultry.

Compared to mammals, poultry appear to have a greater ability to detoxify alkaloids (27). Mainka et al. (45) reported that ergot did not cause changes in weight gain of 28-day-old chickens fed *ad libitum* with an ergot content of 0, 0.5, 1, 2, and 4 g/kg diet. The same levels of ergot reduced weight gain in piglets. Chickens rapidly turn over epithelial cells (within 48 h) that may explain their rapid detoxification of ergot (79, 80).

However, even for poultry long-term exposure to alkaloids may lead to loss of appetite, increased thirst, diarrhea, vomiting, and weakness (81). Similarly, Dänicke (82) exposed Peking ducks to four different diets containing 1, 10, 15, and 20 g ergot/kg diet, respectively. This corresponded to total ergot alkaloid contents of 0.0, 0.6, 7.0, 11.4, and 16.4 mg/kg. They found that feed intake decreased up to 47% with the high ergot diets. While Mainka et al. (45) identified no adverse effects on weight gain of chickens, Dänicke (82) observed a significant growth reduction after 2 weeks, suggesting that existing ergot alkaloid limits for poultry(1 g ergot/kg unground cereal grains in EFSA regulations) may not offer sufficient protection for ducks. Furthermore, Dänicke (82) detected alkaloid residues in edible tissue (5 ng/g) of Peking ducks that also had ergonovine in bile (40 ng/g). Thus, the negative performance of ducks when exposed to 0.6 mg/kg of ergot alkaloids indicates that not all species of poultry are equally tolerant of dietary ergot.

### Impact on the Plant and Animal Industries

Most mycotoxins that infect growing crops and stored feed will be detected based on the type of symptoms shown by livestock (83). However, with ergot displaying broad symptoms, such as heat stress, reduced growth, and feed refusals, producers are challenged to identify the occurrence of ergot toxicosis before it has already had a negative impact on the economics of livestock production. With no universal standard for the safe concentration of ergot in feed, producers must exercise caution when introducing potentially contaminated feed sources such as grain screenings into their feeding programs.

While some livestock can tolerate greater concentrations of ergot in feed, the potential for residual toxins to remain in tissues of animals could cause detrimental effects to the human population (1, 45, 50). More importantly, by-products, such as screenings for livestock feed may be highly contaminated with mycotoxins and, moreover, have a greater potential of harming livestock (57). With the prevalence of ergot increasing from 0.01% in 2002 to 0.025% in 2014 in western Canada (84), it is evident that monitoring ergot is becoming more essential for the safety of both livestock and humans (23).

The need to produce cereal varieties that are capable of withstanding ever-changing climatic conditions has seen an increased use of hybrid varieties of rye and perennial rye breeds in the last 10 years, particularly in European countries such as Germany (10). However, today with grain-cleaning procedures now capable of removing up to 82% of ergot bodies from unprocessed grain (broken ergot sclerotia are less reliably removed as the particle size is similar to the grain), it is evident that improvements are being made, though often at substantial cost to the producer (10, 27).

The European Food Safety Authority (27) suggested that in order to successfully reduce the risk of ergotism in livestock, contaminated cereal grains should undergo seed cleaning, in combination with the adoption of certain husbandry measures such as crop rotation and grazing during summer months to reduce the establishment of flower-heads. However, when considering the level of contamination in cereal crops, it is important to determine alkaloid epimers, as these could alter the toxicity of ergot and cause more harm to livestock than anticipated (10, 37).

Economic impacts surrounding reproductive losses and lowered growth performance are detrimental both on a domestic basis and a global basis. Moreover, with no current treatment marketed to improve symptoms of ergot toxicity and the difficulty of diagnoses, the only available response is to remove the contaminated feed from the diet and allow the liver to detoxify consumed alkaloids (28). It is evident that further investigations are needed to develop effective measures to prevent ergot toxicity in livestock and reduce the economic impact of ergot on agricultural commodities.

## Detoxification and Absorption of Ergot Alkaloids

Livestock and poultry have the capacity to detoxify ergot alkaloids in the liver. However, given the diversity of ergot alkaloids, it is impractical to estimate the length of time required for detoxification and clearance of all alkaloids from the liver. Moubarak et al. (85) characterized the role of cytochrome P450 3A (CYP3A) subfamily in the metabolism of ergot alkaloids, in beef liver microsomes. Ergotamine was metabolized by CYP3A after 60 min of incubation; however, other alkaloids, such as ergocryptine and ergocornine, inhibited CYP3A activity. Cattle intravenously administered ergopeptine rapidly cleared it from the blood through biliary excretion, whereas lower molecular weight alkaloids, such as ergovaline, were excreted in urine (29).

If absorption varies among alkaloids, it may be possible that not all ergot alkaloids are harmful to livestock. Schumann et al. (58) identified that while ruminants have the potential to detoxify mycotoxins in the rumen, microbes are influenced by the passage rate of feed. Increased feed intake reduces feed retention time in the rumen and increases passage rate, impacting digestion and metabolism. Increasing ergovaline in feed from 0, 1.5 to 3 mg/kg diet depressed feed intake, in addition to reducing ruminal and total tract organic matter and neutral detergent fiber (NDF) digestibilities in sheep. This may have lowered the metabolism of ergot alkaloids in the rumen (58, 86). Westendorf et al. (87) reported that feeding 945 mg/d ergovaline (16 mg/kg body weight) decreased DM and NDF ruminal digestibilities, while exposing sheep to 2,346 mg/day ergovaline increased DM and NDF ruminal digestibilities, also possibly due to reduced intake and a longer retention time of feed in the rumen.

Absorption of alkaloids occurs primarily in the ruminant forestomach, with rumen tissue having the greatest transportation rate [25% more than the omasum; (88)]. Extensive excretion of toxins via the urine was noted in steers exposed to infested tall fescue as measured by ELISA (89). In comparison, fecal excretion was limited to 5% of alkaloids fed to sheep, emphasizing the high level of absorption that occurs in ruminants (87). Furthermore, varying differences in liver enzyme function and individual rumen microorganisms will alter an individual animal’s capability of detoxifying alkaloids, leading to varying levels of tolerance (90, 91).

Veterinary recommendations suggest that ergotism can be controlled through an immediate change to an ergot-free diet. However, for pregnant livestock and in particular for sows in late gestation (<1 week prior to parturition), agalactia syndrome cannot be corrected (71). Agalactia syndrome from fescue sources can be corrected in horses through administration of dopamine D2 antagonist domperidone (1.1 mg/kg for 10–14 days). In cases where livestock have been clinically diagnosed with peripheral gangrene, the removal of ergot-contaminated feed will not lead to recovery.

## Technologies and Practical Measures Designed to Reduce the Impact of Ergot on Livestock

### Genetic Engineering Strategies

It is possible to select for genetic resistance to ergot among grain crops, although genetic engineering strategies and the selection of hybrids naturally resistant to molds could be a means of controlling ergot in wheat (92). Though minimal information is known on the role of insects in ergot epidemiology, there is future potential for plants to be selected that deter insects and reduce the spread of mycotoxins (57, 93).

### Development of Vaccines and/or Alkaloid Binders to Allow the Animal to Systemically Bind the Toxic Alkaloids

The development of vaccines against ergot alkaloids is a possible long-term solution. Filipov et al. (94) observed a greater average daily gain (13.0 g/day) when rabbits were vaccinated with 50 μg lysergol-human serum albumin compared to non-treated rabbits (12.1 g/day). While this study evaluated a vaccine against the effects of alkaloids from tall fescue (total dietary alkaloids 340 ppb), development of a vaccine associated with grain alkaloids should also be possible.

Deoxynivalenol is a mycotoxin causing similar symptoms to ergot in livestock, such as reduced feed intake and body weight gain (57). Although ergot alkaloids and DON differ in chemical structure, studies conducted using DON have relevance for ergot. Young et al. (95) revealed that feeding swine corn contaminated with 7.2 mg DON/kg resulted in a reduction in feed intake. When corn was treated with sodium bisulfite, impacts of DON decreased 10-folds. Treatment with sodium bisulfite appeared to remove short-term toxic effects on pigs due to the presence of DON in their diet. Accordingly, there is a possibility that chemical treatments could be developed to reduce the toxicity of ergot alkaloids in feed.

It is also plausible that using alkaloid binders will decrease bioavailability of ergot alkaloids. However, studies of alkaloid binders are limited and in the one published study, Friend et al. (96) evaluated a chemical binding agent, polyvinyl-pyrrolidone (Antitox Vana^®^) and ammonia carbonate and noted that these binders did not reduce the negative impacts of DON on swine production. Further investigations using alkaloid binders to reduce the toxicity of ergot-contaminated grain are required, but care must also be taken to ensure that such binders do not reduce overall nutrient availability (24, 97).

### Isolation of Anaerobic Bacteria to Degrade Ergot Alkaloids before Systemic Absorption

Anaerobic microbes present in the rumen of sheep and cattle are capable of detoxifying some ergot alkaloids and inoculating other microbes into the rumen might be beneficial in this regard. Anaerobic microbes in the gut of the red wiggler earthworm, *Eisenia fetida*, degraded over 60% of ergovaline, with the flora responsible for this degradation from four major phyla: *Plantomyce, Chloroflexi, Bacteroides*, and *Proteobacteria* (16). Further research to isolate and characterize microorganisms that are capable of detoxifying ergot alkaloids may allow their use as a direct-fed microbial to minimize the impact of feed ergot on animals.

### Hydrothermal Treatment Effects on Ergot Alkaloid Content in Contaminated Grain

Hydrothermal treatments are often incorporated to improve the digestibility of nutrients and feed value, particularly for non-ruminant species (45). Treating ergot-contaminated grain with steam for 2 min at 95°C at 17% moisture, followed by 5 s at 120°C at 18% moisture decreased total alkaloid content by 10%, with reductions becoming more marked with increasing levels of alkaloids (45). This method could be employed during the feed processing stage to further reduce alkaloids, although impacts on alkaloid toxicity would require investigation prior to use in livestock feeds.

### Other On-Farm Prevention Measures

Irrespective of advanced technologies that can be potentially implemented on-farm to minimize negative impacts, ergot toxicoses are mainly controlled by limiting ergot presence at all levels of production, including storage, milling, and delivery (57). Chemical treatments used to clean the grain kernels can be implemented to significantly reduce the toxin level if the ergot contamination is not too severe. Removal of grain dust and lighter, shriveled kernels through density segregation can also reduce the risk of ergot poisoning (5, 23). Other procedures, such as soaking, dehulling, roasting, or high velocity air cleaning of grain can be used to remove surface ergot contaminants (27).

A variety of prevention measures have been identified to help producers minimize ergot establishment and growth in cereal crops (5) including:

(a) Limiting the number of damaged kernels from birds and insects, as molds thrive on kernels where the pericarp or hull has been compromised.
(b) Harvesting grain as soon as practically possible, especially when ergot is visually detected. Areas on-farm highly susceptible to ergot should be harvested as forage prior to the heading stage in order to avoid the formation of ergot bodies.
(c) Correctly storing and drying grain. With high moisture content, conditions remain anaerobic, increasing the likelihood of mycotoxin contamination.
(d) Rotating crops to avoid the carry-over of molds, as sclerotia are capable of remaining viable prolonged periods. Increasing seedling vigor and using seeds treated with fungicides will reduce seed-borne inoculum.

## Conclusion

Minimizing the economic loss of producers due to ergot contamination in grains and subsequent ergot toxicoses in livestock is challenging. The diversity of fungal species and ergot alkaloids, their interactions with the surrounding environments for different crops and their varying toxicities in different tissues and/or livestock and poultry add to the complexity of the issue. As the climate is changing to favor ergot-producing fungi in some parts of the world and as regulations for human food become stricter, the frequency of ergot-contaminated grains will likely increase in the future. Accordingly, strategies to reduce risks of ergot toxicoses are required to support the livestock industry. Although regulations and recommendations for the ergot alkaloid level in animal feed exist, a scientific basis for these recommendations is generally lacking.

While eliminating the threat of ergot toxicoses in livestock is likely impossible, application of some practical measures, including chemical cleaning grain, would minimize their impact, but the process is costly and may leave toxic residues. Devising methods to combat toxicoses could be aided by a better understanding of the physiological pathways impacted by ergot alkaloids. Moreover, experiments incorporating individual alkaloids in *in vitro* and *in vivo* animal studies would benefit this effort. Alkaloid binders and the use of antioxidants to lessen the effects of ergot poisoning would be valuable if effective binders could be identified. With grain contamination by ergot increasing annually and globally, effective new technologies are required to either reduce the occurrence of the ergot in grains or reduce the toxicity of alkaloids for livestock.

## Author Contributions

SC-M collected literature and wrote the manuscript; TM, KS, and YW perceived the concept and co-wrote the manuscript; BB, JM, and AC performed the critical review and co-wrote the manuscript.

## Conflict of Interest Statement

The authors declare that this review was prepared in the absence of any commercial or financial relationships that could be construed as a potential conflict of interest.
